# Adenylyl Cyclase Plays a Regulatory Role in Development, Stress Resistance and Secondary Metabolism in *Fusarium fujikuroi*

**DOI:** 10.1371/journal.pone.0028849

**Published:** 2012-01-26

**Authors:** Jorge García-Martínez, Attila L. Ádám, Javier Avalos

**Affiliations:** 1 Department of Genetics, Faculty of Biology, University of Seville, Seville, Spain; 2 Mycology Group of the Hungarian Academy of Sciences, Agricultural Biotechnology Center, Institute of Plant Protection, Szent István University, Gödöllő, Hungary; University of Missouri-Kansas City, United States of America

## Abstract

The ascomycete fungus *Fusarium fujikuroi* (*Gibberella fujikuroi* MP-C) produces secondary metabolites of biotechnological interest, such as gibberellins, bikaverin, and carotenoids. Production of these metabolites is regulated by nitrogen availability and, in a specific manner, by other environmental signals, such as light in the case of the carotenoid pathway. A complex regulatory network controlling these processes is recently emerging from the alterations of metabolite production found through the mutation of different regulatory genes. Here we show the effect of the targeted mutation of the *acyA* gene of *F. fujikuroi*, coding for adenylyl cyclase. Mutants lacking the catalytic domain of the AcyA protein showed different phenotypic alterations, including reduced growth, enhanced production of unidentified red pigments, reduced production of gibberellins and partially derepressed carotenoid biosynthesis in the dark. The phenotype differs in some aspects from that of similar mutants of the close relatives *F. proliferatum and F. verticillioides*: contrary to what was observed in these species, Δ*acyA* mutants of *F. fujikuroi* showed enhanced sensitivity to oxidative stress (H_2_O_2_), but no change in heavy metal resistance or in the ability to colonize tomato tissue, indicating a high versatility in the regulatory roles played by cAMP in this fungal group.

## Introduction

Some species of the ascomycete fungus *Fusarium* are useful research models for secondary metabolite production. A representative example is *Fusarium fujikuroi*, able to produce gibberellins, carotenoids or bikaverin, among other compounds of potential biotechnological interest [1]. The gibberellins are growth-promoting plant hormones with applications in agriculture [2]. Among them stands out for its physiological effects gibberellic acid (GA3), the major gibberellin product in this fungus. Carotenoids are fat-soluble pigments produced by photosynthetic organisms and many heterotrophic bacteria and fungi [3]. In plants they function as accessory pigments of the photosynthetic machinery [4] and in animals they are the source of physiologically important apocarotenoids, e.g., retinal and retinoic acid [5]. *F. fujikuroi* produces the orange apocarotenoid neurosporaxanthin (β-apo-4′-carotenoic acid) and minor amounts of other carotenoids [6], including β-carotene and, presumably, retinal [7]. Gibberellins and carotenoids are mevalonate-derived terpenoids produced from the same precursor, geranylgeranyl pyrophosphate, although their synthesis occurs in different cell compartments [8]. Bikaverin is a red polyketide pigment with antibiotic properties against protozoa and fungi [9], whose synthesis begins with the condensation of acetate units.

The biochemistry and regulation of these biosynthetic pathways has received considerable attention. The genes needed for the synthesis of gibberellins [10] and bikaverin [11] are organized in clusters. The genes required for retinal synthesis also are clustered [12], [13], but two additional genes needed for neurosporaxanthin biosynthesis are located elsewhere in the genome [14], [15]. The respective gene sets for gibberellin, bikaverin and carotenoid biosyntheses are subject to different coordinated regulations in response to environmental signals. The three pathways have in common their activation by nitrogen starvation, although some differences are found in their particular responses [16]. Additionally, they respond to other regulatory signals in a pathway specific manner: e.g., carotenoid biosynthesis is induced by light [17] and bikaverin production requires acidic pH [18]. Different regulatory proteins are involved in these responses, such as AreA in nitrogen regulation [19], and PacC in pH regulation [11]. The mutation of the gene for the White Collar protein WcoA, predicted to mediate light induction of carotenogenesis, did not impede such photoresponse, but altered the regulation of bikaverin and GA biosyntheses [20]. Recent data showed the participation of other proteins in the regulation of these biosynthetic pathways, such as those from the Velvet complex [21], formerly identified in *Aspergillus nidulans* [22].

The increasing number of influential proteins points to a complex regulatory network involved in the control of secondary metabolism in *F. fujikuroi* in connection with other physiological and developmental processes. A recent example of such regulatory complexity was provided by the deregulation of bikaverin biosynthesis in mutants of adenylyl cyclase in *F. proliferatum* [23] and *F. verticillioides* [24]. This enzyme, whose activity is regulated by the Gα subunits of heterotrimeric G proteins in response to ligand-activated G-protein-coupled receptors, mediates the synthesis of cAMP, a chemical signal involved in the control of a diversity of fungal processes. Mutants lacking a functional adenylyl cyclase usually exhibit alterations in growth and development, but the severity of the alterations differs between different fungi. A representative example is *Neurospora crassa*, where such mutation leads to the “crispy” phenotype, externally characterized by retarded growth, reduced aerial development and premature conidiation [25]. The morphological alterations produced by lack of cAMP synthesis also involve other developmental stages of fungal life cycles: as two representative examples, mating and pathogenesis are affected in *Cryptococcus neoformans* [26] and *Ustilago maydis* [27]. Moreover, virulence is usually diminished or abolished in pathogenic species. E.g. formation of invasive appressoria is impaired in *Magnaporthe grisea* [28], aberrant sclerotia are formed in *Sclerotinia sclerotiorum* [29] and switch from yeast to hyphal growth is severely affected in *Candida albicans* [30].

The adenylyl cyclase mutation has a highly pleiotropic phenotype in *F. proliferatum* and *F. verticillioides*. In addition to bikaverin deregulation, mutants exhibit reduced vegetative growth rate, delayed microconidia germination, alterations in micro- and macroconidia production, enhanced tolerance to abiotic conditions, such as heat shock or oxidative stress, and reduced pathogenic capacity [23], [24]. Furthermore, female fertility and vegetative self-incompatibility were suppressed in *F. proliferatum* [23]. However, in contrast to the effect on bikaverin production, lack of adenylyl cyclase produced no change in the biosynthesis of another polyketide toxic metabolite, fumonisin B1, in any of these species. Here we describe the effect of the adenylyl cyclase mutation on development, resistance to stress conditions, and secondary metabolism in *F. fujikuroi*. The mutants of the gene, that we called *acyA*, produced lower amounts of gibberellins and showed alterations in the pigmentation pattern. As expected, deletion mutants of this gene exhibited a decreased growth rate and altered conidiation, but in contrast to former *Fusarium* reports, they were more sensitive to oxidative stress and were not affected in their resistance to heavy metals or their capacity to colonize tomato fruit tissue.

## Results

### Generation of *F. fujikuroi* Δ*acyA* mutants

**Figure 1:**
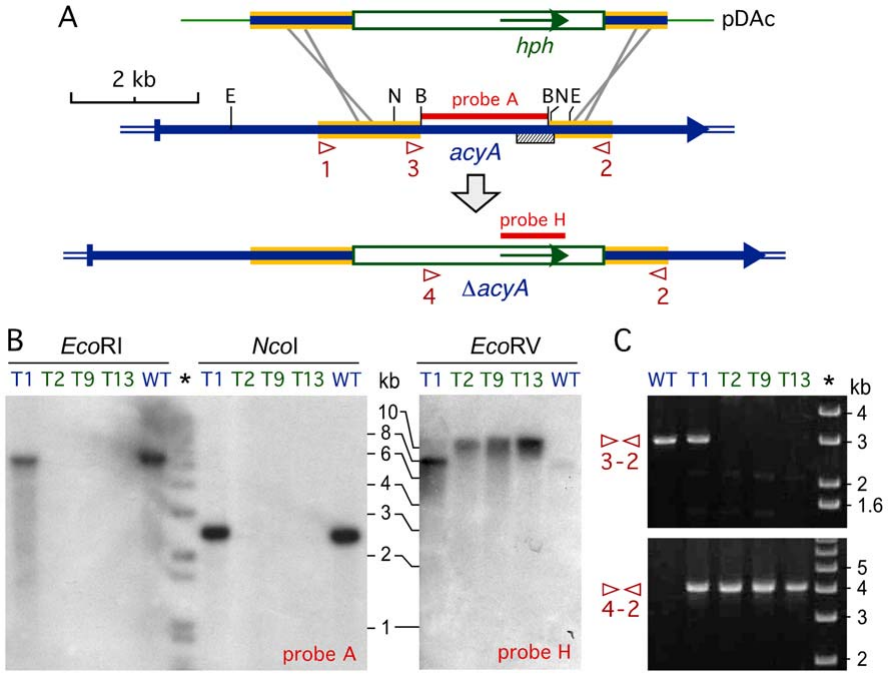
Generation of Δ*acyA* mutants of *F. fujikuroi*. A: Schematic representation of the gene replacement event leading to the Δ*acyA* mutation. The thick arrow represents the *acyA* gene. Segments shaded in yellow in the background, derived from the original 4.5 kb *acyA* PCR fragment, support the homologous recombinations leading to the replacement. The white box is the HygR cassette, containing the *hph* gene (green inner arrow). The *Bam*HI-delimited segment, absent in the mutants, was used as a probe in the Southern blot experiment shown below (probe A). The striped box shows the predicted location of the catalytic domain. Small arrowheads indicate the position and orientation of primers 1 and 2, used to obtain the *acyA* PCR fragment, and primers 3 and 4, used with primer 2 in the PCR experiment displayed below. B: Southern blots of genomic DNA from the wild type and four transformants digested with *Eco*RI or *Nco*I and hybridized with an internal *acyA* DNA segment (Probe A) or digested with *Eco*RV and hybridized with the *hph* gene of the hygR cassette (Probe H). Probe locations and relevant *Eco*RI (E) or *Nco*I (N) sites are indicated on the upper scheme. The represented DNA segments lack *Eco*RV sites. Here and in the right panel, the star indicates the lane of size markers; relevant sizes in kb are indicated on the right. C: Agarose gel electrophoresis of amplification products obtained by PCR from DNA samples of the same strains with the primer sets indicated on the left (see upper scheme for primer locations). Lack of 3 kb DNA segment with primers 3 and 2 reveals the absence of the wild type *acyA* gene. Amplification of the 4 kb DNA segment with primers 4 and 2 reveals the presence of a hygR-interrupted *acyA* gene.

BLAST queries of the FpACY1 adenylyl cyclase protein sequence from *F. proliferatum* ([23], GenBank accession DQ067619) against the publicly available *Fusarium* genome databases, *F. oxysporum*, *F. verticillioides* and *F. graminearum*, identified a single gene for this protein (FVEG01363, FGSG01234 and FOXG00154, respectively). So, we predicted the occurrence of a single homologous gene in *F. fujikuroi*, that we named *acyA*. A primer set designed from conserved sequences of the four orthologous *Fusarium* adenylyl cyclase genes (primers 1 and 2, Fig. 1A), allowed the amplification and cloning of a 4.5 kb DNA segment of *acyA* of *F. fujikuroi*, containing the catalytic domain of the enzyme (Fig. 1A, [23]). In support of the occurrence of a single *acyA* gene, a single hybridizing band was observed in Southern blot analysis when digested wild type *F. fujikuroi* genomic DNA was probed with an internal segment of the gene (Fig. 1B).

To obtain null mutants of *acyA*, a plasmid (pDAc) was constructed in which an internal *Bam*HI 2 kb segment, containing most of the catalytic domain, was removed from the cloned 4.5 *acyA* fragment and replaced with a *hygR* cassette (Fig. 1A). Transformation of the wild type strain FKMC1995 with linearized pDAc led to the isolation of 13 hygromycin-resistant transformants. These strains were passed three times through single conidia on hygromycin-supplemented medium to ensure homokaryosis, and 11 of them conserved the resistant phenotype. Visual inspection of the colonies revealed that 5 transformants exhibited the wild type phenotype, while 6 grew slower and became pigmented upon prolonged incubation. These alterations coincided with those formerly described for the adenylyl cyclase mutants of *F. verticillioides* and *F. proliferatum* [23], [24], and so they were suspected to have lost a functional *acyA* gene. Three of such transformants, T2, T9 and T13, were chosen for detailed molecular characterization in comparison to the wild type and a wild type-like transformant, T1. Southern blot and PCR analyses confirmed the replacement of the wild type *acyA* allele with the *hph*-interrupted sequence in the three altered strains (Fig. 1B and C), referred hereafter as Δ*acyA* mutants. Transformant T1 had presumably inserted ectopically the pDAc DNA by non-homologous recombination, as indicated by the simultaneous detection of the wild type allele and the interrupted sequence. This transformant was used as an additional *acyA* ^+^ control in subsequent phenotypic analyses.

### The Δ*acyA* mutants show alterations in growth, morphology and conidiation

**Figure 2:**
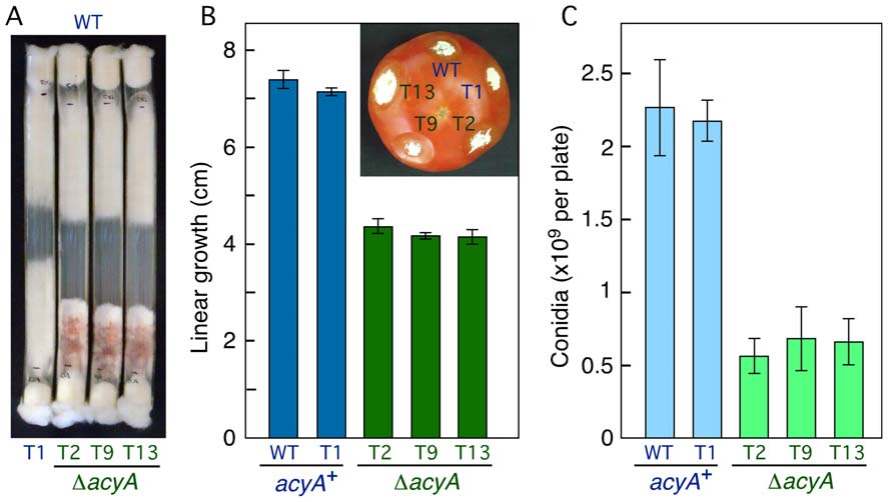
Growth and conidiation of the *acyA* ^+^ and Δ*acyA* strains. A: Growth of the four transformants against the wild type in race tubes after 21 days incubation at 30°C in DGasn minimal medium. B: Average and standard deviation of linear growth of the wild type and the four transformants in race tubes after 21 days. Inner picture: ability of the five strains to colonize tomato fruit. C. Average and standard deviation of conidia production by the same strains on Petri dishes incubated for 8 days in the dark. Data from two independent experiments.

Incubation of the three Δ*acyA* mutants under different growth conditions confirmed phenotypic alterations, not exhibited by parallel cultures of the control strains. Experiments carried out in race tubes showed a 35% reduction in linear growth compared to the strains carrying an intact *acyA* gene (Fig. 2A and B). Upon aging, the Δ*acyA* mutants exhibited a reddish pigmentation in the race tubes (Fig. 2A), a trait described in a later section. Additionally, surface cultures of the mutants produced fewer conidia (Fig. 2C). This difference cannot be attributed to the slower mycelial development, since these analyses were carried out on surface colonies that stopped their growth approximately four days before conidia determination. In a former experiment in carboxymethyl cellulose supplemented medium [31], the *acy1* mutants of *F. proliferatum* produced more conidia than the wild type [23]. However, incubation of the same strains under the culture conditions tested for *F. fujikuroi* revealed the same conidiation pattern in both species (data not shown). Thus, the *acyA* gene plays a central role in mycelial development, including conidia formation. However, in contrast to *F. proliferatum* [23], the reduced growth capacity did not affect the ability of the mutants to colonize tomato fruit tissue (inset picture in Fig. 2B).

**Figure 3:**
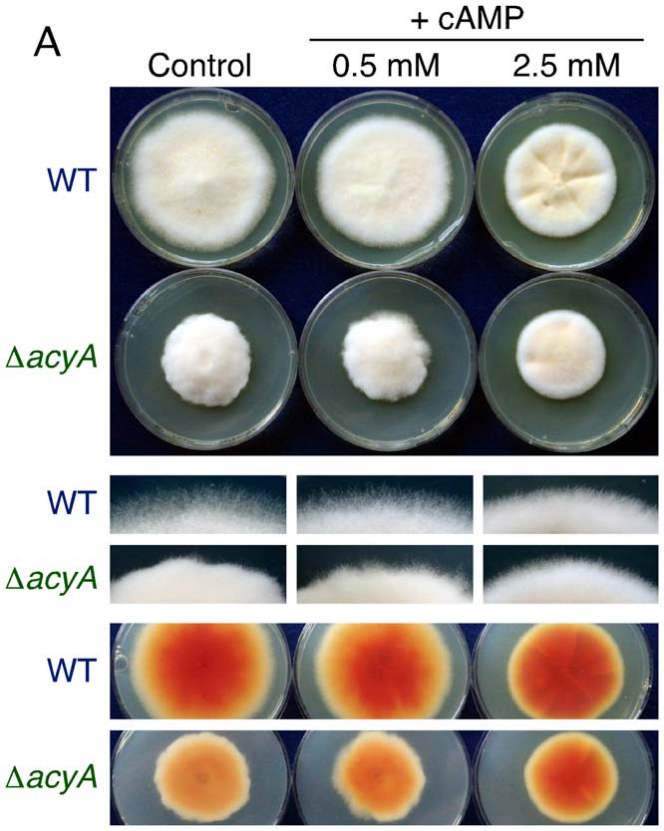
Effect of cAMP addition on growth and pigmentation of the wild type and an Δ*acyA* strain. A: Colonies of the wild type and the Δ*acyA* mutant T9 after eight days incubation at 30°C on DGasn minimal agar (5.5 cm Ø Petri dishes) supplemented with the indicated concentration of the cAMP analogue dibutyryladenosine 3′,5′-cyclic monophosphate. Middle pictures: detail of colony edges from the same cultures. Below: pigmentation of the back of the colonies.

Addition of exogenous cAMP to agar cultures of a Δ*acyA* mutant did not fully restore normal growth (Fig. 3). However, growth and morphology of the wild type was visibly altered at the higher cAMP concentration tested, resembling those of the Δ*acyA* mutant under the same conditions. Therefore, the phenotypic differences between wild type and Δ*acyA* mutant were attenuated by cAMP addition. This was particularly manifest at the colony edges, sparse in the wild type and dense in the compact Δ*acyA* colonies in control plates, but with a similar aspect for both strains in the presence of 2.5 mM cAMP (Fig. 3). Furthermore, an orange pigmentation unrelated to carotenoids, manifest on the back sides of wild type colonies grown in the dark at 30°C, was much weaker in the Δ*acyA* mutant but it was restored in 2.5 mM cAMP supplemented medium (Fig. 3). These observations strongly suggest that the phenotypic alterations exhibited by the Δ*acyA* mutants are due to lack of cAMP synthesizing activity.

### The Δ*acyA* mutants are affected in stress sensitivity

The adenylyl cyclase mutants of *F. proliferatum* and *F. verticillioides* also exhibited alterations in their responses to different stress conditions, such as heat-shock, oxidative agents, or heavy metals [23], [24]. We examined the effect of these stresses on the *F. fujikuroi* mutants compared to the control strains. First, we tested the ability of the Δ*acyA* and *acyA* ^+^ strains to grow under toxic concentrations of two heavy metals, Cd and Cu. Growth inhibition, referred to control plates without the metal, was similar irrespective of the presence of a functional *acyA* gene in the tested strain (Figure S1).

**Figure 4:**
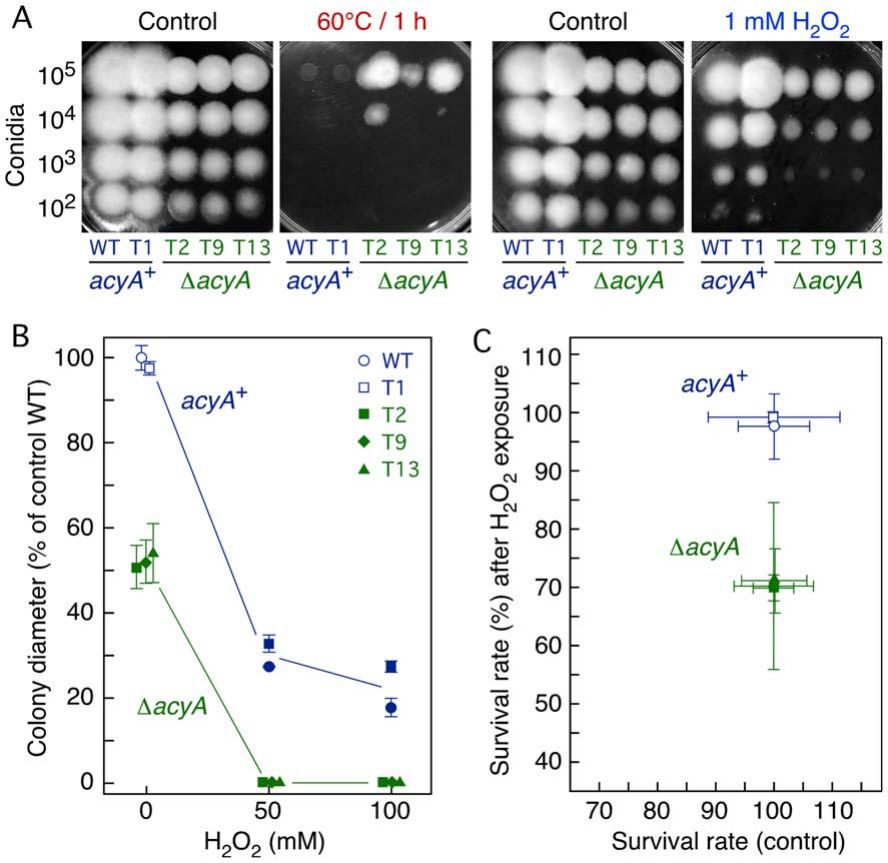
Effect of the Δ*acyA* mutation on growth or viability under heat or oxidative stress. A: Serial conidia-dilution tests of the effect of heat stress (60°C/1 h) and oxidative stress (1 mM H_2_O_2_) on DGasn agar plates. Each spot was inoculated with the conidia amounts indicated on the left. B: Effect of H_2_O_2_ concentration (50–100 mM) on radial growth of the *acyA* ^+^ and Δ*acyA* strains on DGasn agar plates. Overlapping symbols were separated for better visualization. C: Survival of conidia following 2 h incubation in liquid DGasn medium supplemented with 5 mM H_2_O_2_.

One hour exposure to 42°C, 45°C or 50°C did not affect significantly the growth capacity of the Δ*acyA* mutants compared to the *acyA* ^+^ strains in conidia-dilution tests on DGasn agar plates. However, one hour exposure to 60°C completely abolished the growth of the control strains, while the Δ*acyA* mutants were still able to grow with 10^5^ conidia (Fig. 4A). In this experiment the cells were exposed to 60°C immediately after inoculation of the conidia drops, but similar results were obtained if the 60°C treatment was done 4 h after inoculation, i.e., when the germination process was already triggered (data not shown). Hence, cAMP probably participates in the tolerance mechanism against heat shock in a similar manner than in other *Fusarium* species. Former studies in *F. verticillioides* and *F. proliferatum* revealed 49°C and 45°C as effective heat shock temperatures in liquid media assays, respectively. The comparison of heat shock sensitivity of wild type *F. fujikuroi* and *F. proliferatum* in conidia-dilution assays on DG agar plates (both species were sensitive to heat stress only at 55–60°C in a conidial concentration dependent fashion) suggest that this disparity is due to differences in culture conditions rather than to higher heat tolerance of *F. fujikuroi* (data not shown).

Similar conidia-dilution tests achieved with a low (1 mM) H_2_O_2_ concentration showed a reduction in the growing capacity of the five *F. fujikuroi* strains, but the inhibition was apparently more pronounced in the Δ*acyA* mutants (Fig. 4A). Therefore, we decided to investigate in more detail the effect of this oxidative agent. Incubation of the strains on DGasn agar plates supplemented with higher H_2_O_2_ concentrations confirmed a greater sensitivity of the Δ*acyA* mutants: they were not able to grow at either 50 or 100 mM H_2_O_2_, while the strains with the functional *acyA* allele still showed a significant growing capacity (Fig. 4B). In another approach, conidia from the same strains were exposed for two hours to 5 mM H_2_O_2_ in DGasn minimal liquid medium and tested for their ability to develop colonies after plating on CM agar. As a result, the Δ*acyA* mutants produced about 30% less colonies than the control strains, which were not significantly affected by this treatment (Fig. 4C). We conclude that lack of adenylyl cyclase in *F. fujikuroi* results in a weakened response to H_2_O_2_-induced oxidative damage.

### The Δ*acyA* mutants overproduce a red pigment

**Figure 5:**
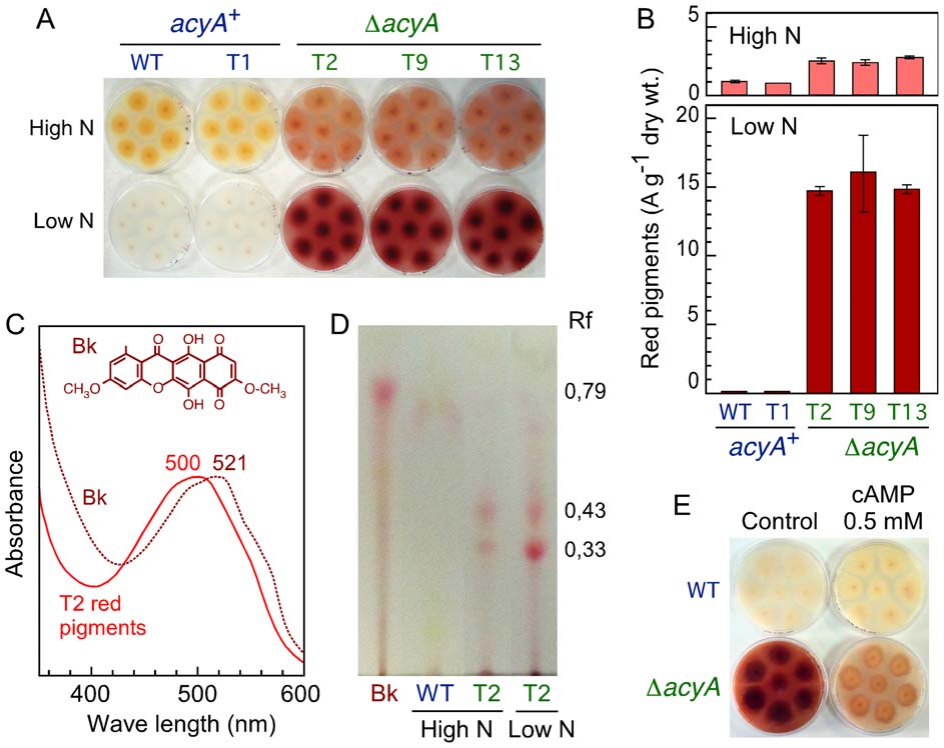
Production of red pigments by the *acyA* ^+^ and Δ*acyA* strains. A: Agar cultures of the *acyA* ^+^ and Δ*acyA* strains grown for 14 days at 30°C on high or low nitrogen medium. B: Concentration of red pigments in the mycelia from the plates displayed on the left picture. The data show absorption units corrected by dry weight of the samples, and referred to the absorption of the wild type grown in high nitrogen medium, which was taken as 1. C: Absorption spectra of the red pigments isolated from the T2 mutant grown in high nitrogen medium compared to the spectrum of our bikaverin standard (Bk, dashed line). Chemical structure of bikaverin is shown above. D: TLC separation of the bikaverin standard (Bk) and the red pigments produced by the wild type on high nitrogen medium and by the T2 mutant on high or low nitrogen medium. E: Effect of addition of 0.5 mM cAMP (dibutyryladenosine 3′,5′-cyclic monophosphate) on pigmentation in agar cultures of the wild type and the Δ*acyA* strain T9 grown for 14 days at 30°C on low nitrogen medium.

As mentioned above (Fig. 2A), the Δ*acyA* mutants exhibited a reddish pigmentation, absent in the control strain. This color was more apparent under nitrogen starvation, and it diffused to the culture medium (Fig. 5A), indicating that the pigment is soluble and secreted out of the cell. The enhanced reddish pigmentation fits the derepressed bikaverin production formerly found in the adenylyl cyclase *mutants* of *F. proliferatum* and *F. verticillioides* [23], [24]. The production of this pigment was investigated in the five strains under analysis, grown on minimal agar with either high or low nitrogen content (Fig. 5B). According to absorption data, the two control strains produced low pigment amounts in high nitrogen agar, while the production by the three Δ*acyA* mutants increased about three-fold. The difference was more noteworthy in low nitrogen agar, where the Δ*acyA* mutants produced high pigment amounts while only traces were found in the cultures of the control strains. The accumulation of the pigment was drastically reduced in the presence of exogenous cAMP (Fig. 5E), providing further evidence for the lack of cAMP as the major cause for the phenotypic alterations of the Δ*acyA* mutants.

The absorption spectra of the red pigments extracted from the Δ*acyA* mutants differed from that of bikaverin, as deduced from comparison with a bikaverin sample produced by the strain SG1 [16]. While the latter coincided with the published bikaverin spectrum [32], including maximal absorption wavelength (521 nm), the spectrum of the red pigment produced by the Δ*acyA* mutants had a maximal absorption at *ca*. 500 nm (Fig. 5C). Absorption spectra of the pigments from the control strains, produced in much lower concentrations, were more similar to that of our bikaverin standard (result not shown). To gain more insights on the chemical difference between the pigments accumulated by control and mutant strains, the samples obtained in these analyses were separated on TLC and compared to SG1-produced bikaverin. The pigment from the Δ*acyA* cultures, either in high or low nitrogen agar, showed two bands of different chromatographic properties than bikaverin (Fig. 5D). However, the absorption spectrum and chromatographic separation of the pigment produced by the wild type strain were more similar to those of bikaverin, although it run at a slightly lower position on the TLC plate and it was hardly visible because of its low concentration. The chemical nature of the red pigments produced by the Δ*acyA* mutants is currently under investigation.

### The Δ*acyA* mutants exhibit changes in gibberellin and carotenoid production

**Figure 6:**
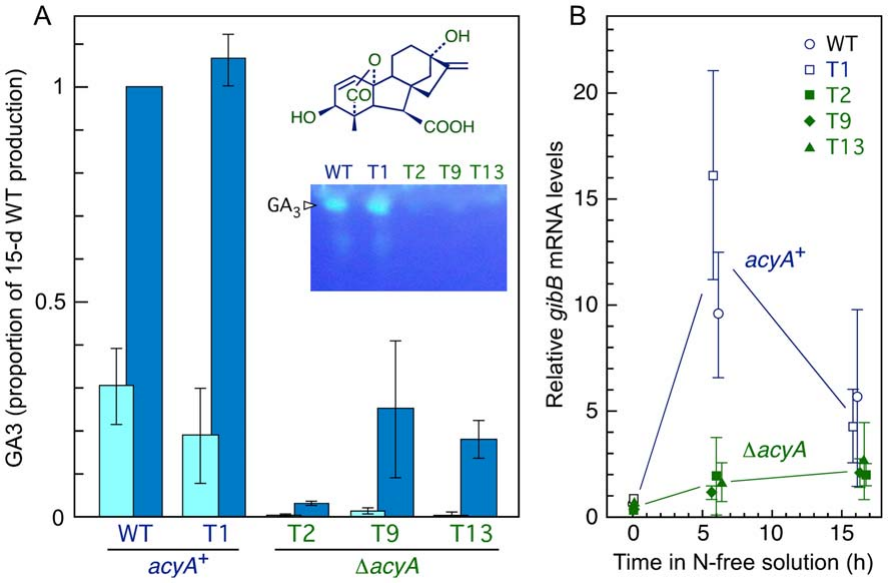
Production of gibberellins by the *acyA* ^+^ and Δ*acyA* strains. A: Relative GA3 contents in the filtrates of the cultures of the indicated strains after 9 days (pale front bars) and 15 days (dark back bars) incubation in low nitrogen broth in the dark. The data show average and standard deviation from two independent experiments. Inner picture: TLC separation of gibberellin samples from the five strains in a representative experiment. The fluorescent spot is GA3 (chemical formula shown above). B: Quantitative RT-PCR analyses of *gibB* mRNA levels in the five strains grown in high nitrogen medium for 3 days and incubated afterwards in 2 g l^−1^ glucose for the time indicated in abscissas.

The effect of the Δ*acyA* mutation on gibberellin and carotenoid biosyntheses was investigated under appropriate culture conditions for each pathway. For gibberellin production, secreted GA3 levels were assayed by TLC and HPLC in shake cultures of the five strains grown in low nitrogen broth. As expected, significant GA3 amounts (about 42 mg l^−1^ after 15 days incubation) were detected in the cultures of control *acyA^+^* strains, but GA3 concentration was severely reduced in the Δ*acyA* cultures (Fig. 6A). To test if the Δ*acyA* mutation affects negatively the induction of the *gib* genes by nitrogen starvation, mycelia of the five strains grown in high nitrogen broth were washed and transferred to a nitrogen-free solution. Upon these controlled inducing conditions, the mRNA levels of gene *gibB*, coding for the key enzyme in GA biosynthesis ent-kaurene synthase/ent-copalyl diphosphate synthase (*cps/ks* [33]), showed a rapid and transient induction. This result, and differences in time-course patterns of gibberellin accumulation [34], suggests regulatory differences with another wild type strain previously investigated, IMI58289, in which the mRNA induction under the same experimental conditions was slower and sustained [16]. In agreement with the lower GA3 production, *gibB* mRNA induction was significantly reduced in the Δ*acyA* mutants compared to the *acyA* ^+^ control strains (Fig. 6B), pointing to cAMP as a positive signal in the regulation of GA biosynthesis.

**Figure 7:**
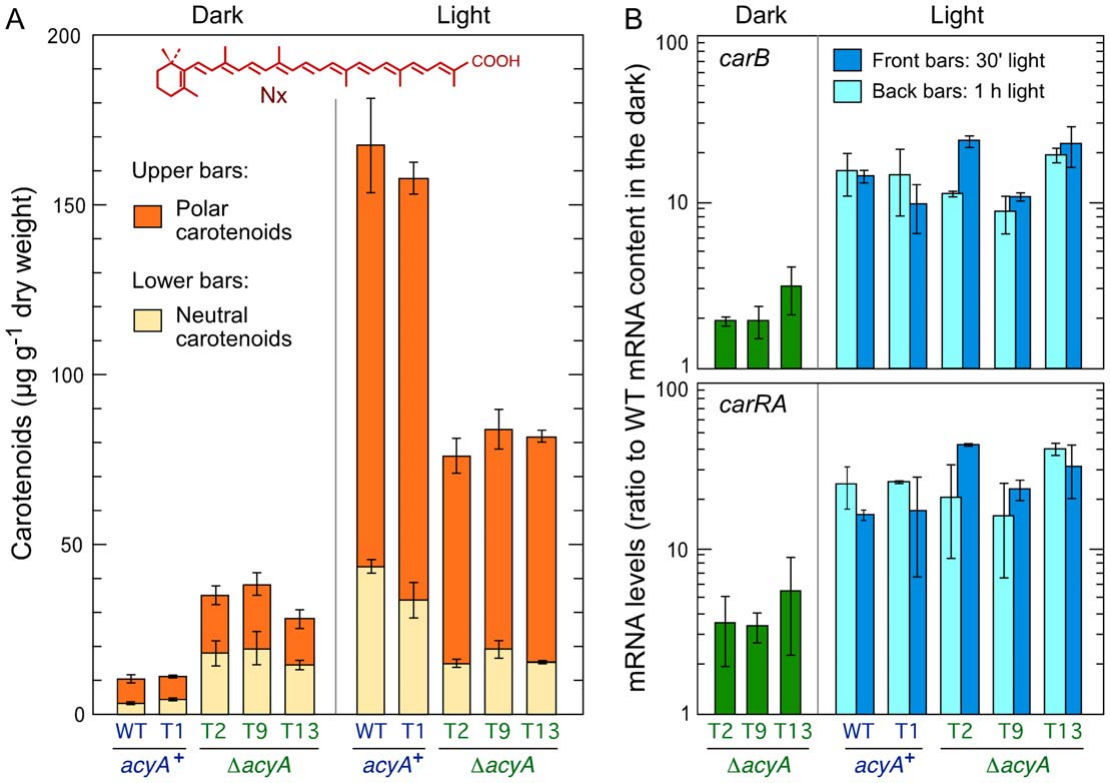
Production of carotenoids and *carRA*/*carB* gene expression in the *acyA* ^+^ and Δ*acyA* strains. A: Carotenoid content in mycelia grown for 7 days on minimal medium in the dark or under continuous illumination. The data show average and standard deviations for the amounts of polar (upper bars) and neutral (lower bars) carotenoids in two independent experiments. The chemical formula of neurosporaxanthin (Nx) is shown above. B: mRNA levels of the enzymatic genes for the carotenoid pathway *carRA* and *carB* in the same strains grown in the dark or illuminated for 30 or 60 min. Data show average and standard deviation from two independent experiments. Relative mRNA levels are referred to the value of the wild type in the dark.

Because of the regulation by light of the carotenoid pathway, the production of these pigments was analyzed on surface cultures, grown either in the dark or under continuous illumination. As expected, the control strains exhibited a marked photoinduction, with a 15-fold increase in the amount of carotenoids in illuminated cultures compared to the dark (Fig. 7A). However, the carotenoid content of the Δ*acyA* mutants was increased about 3-fold in the dark and it was reduced by 50% in the light compared to the control strains. Consequently, the photoinduction rate was markedly reduced in the Δ*acyA* mutants. The ratio of polar (neurosporaxanthin) to neutral carotenoids (precursors) was not significantly modified in the Δ*acyA* mutants, indicating that all the enzymatic activities of the carotenoid pathway were similarly affected by the loss of functional adenylyl cyclase.

For a better understanding of the regulatory basis for the altered carotenoid accumulation, the mRNA levels of two key enzymatic genes of the carotenoid pathway, *carRA* and *carB* [1], were determined in the *acyA* ^+^ and Δ*acyA* strains grown in the dark and upon illumination. The dark levels were induced about 4-fold for *carRA* and 2-fold for *carB* in the Δ*acyA* mutants compared to the wild type (Fig. 7B), fitting their corresponding carotenoid contents *in vivo* (Fig. 7A). In the control transformant T1, *carRA* and *carB* mRNA levels in the dark were even lower than in the wild type (not shown in the graph). However, mRNA levels in the light were similar in any of the strains tested, either after 30 min or 1 h illumination. Therefore, in contrast to the partial deregulation in the dark, the lower carotenoid content of the Δ*acyA* mutants in the light cannot be attributed to changes in *carB* or *carRA* expression.

## Discussion

The product of adenylyl cyclase, cAMP, is a key regulatory signal in all taxonomic groups, from bacteria [35] to mammals [36]. Many reports have attributed biological functions to cAMP in filamentous fungi. The recent finding of the partial deregulation of bikaverin biosynthesis in adenylyl cyclase mutants of *F. proliferatum* and *F. verticillioides* led us to undertake the investigation of a possible regulatory effect on gibberellin and carotenoid production in *F. fujikuroi*. As expected, the targeted mutation of the *acyA* gene in this species also resulted in reduced growth and enhanced reddish pigmentation. However, at least under the experimental conditions tested, the red pigments produced by the Δ*acyA* mutants of *F. fujikuroi* are not bikaverin, as indicated by the significant shift in the maximal absorption wavelength and the different behavior on thin layer chromatography. The different characteristics of these red pigments suggest a novel class of secondary metabolites, whose chemical nature remains to be elucidated.

As observed in other fungi (see, e.g., [24], [37]), addition of exogenous cAMP to adenylyl cyclase mutants did not lead to a total recovery of wild type growth, a result explained by the noticeable toxic effects of this compound. Experiments were done with dibutyryl-cAMP, a cell permeable cAMP analog widely used in research because of its stability and capacity to mimic cAMP activity. The recovery of wild type phenotypic characteristics by the Δ*acyA* strain grown in the presence of dibutyryl-cAMP points to lack of cAMP as the major cause of the mutant phenotype. cAMP participates in a well-known signaling cascade, whose major components are highly conserved in fungi [38]. A key function of this molecule is the activation of cAMP-dependent protein kinases, which in turn act on other downstream elements of the cascade. The phenotypic similarities of adenylyl cyclase and PKA mutants in *F. verticillioides* [24] suggest the participation of cAMP and protein kinase A (PKA) in a similar regulatory cascade in *F. fujikuroi*.

Lack of adenylyl cyclase activity of *F. fujikuroi* resulted in a severe reduction in GA3 content under nitrogen starvation, i.e., the optimal conditions for its production. This is likely due to a diminished transcriptional induction of the biosynthetic genes of the pathway, as indicates the lower mRNA amounts found for gene *gibB* (*cps/ks*, [33]), coding for the enzyme synthesizing the gibberellin precursor, ent-kaurene. A similar down-regulation effect of gibberellin production was observed in other mutants, such as those of the global nitrogen regulator AreA [19], the WC-1 protein WcoA [20], or the global regulator Velvet [21]. The regulatory gene mutated in the *carS* strain SG1, playing a key role in the control of the carotenoid pathway [16], may also be added to this list. The combination of the available data suggest a complex model in which a diversity of regulatory proteins exert specific effects, directly or indirectly, on the expression of the enzymatic genes for the biosynthesis of gibberellins and other secondary metabolites, such as the bikaverin [39]. Our results support a role for cAMP signaling in the regulatory network currently under investigation, in which the number of proteins involved is still expanding. The integration of adenylyl cyclase in this model will require more data, such as the analysis of double mutations with other participating regulatory genes.

The phenotype of the Δ*acyA* mutants of *F. fujikuroi* resembles that of the *velvet* and *wcoA* mutants in their reduced conidiation rates. Alteration of sporulation is a common trait in adenylyl cyclase mutants of formerly investigated filamentous fungi. As expected, the consequence of the mutation was similar in other *Fusarium* species, such as *F. verticillioides* [24] and *F. proliferatum*, or in taxonomically related species, as the ascomycetes *Magnaporthe grisea* [28] and *Aspergillus fumigatus* [40]. However, a higher conidiation activity was observed in adenylyl cyclase mutants of *N. crassa* [25] and, under certain specific culture conditions, of *F. proliferatum* [23]. The latter case demonstrates the dependence of the Δ*acyA* phenotype on the environmental conditions. Likewise, the lower conidiation exhibited by the *wcoA* mutants of *F. fujikuroi* compared to the wild type was reversed under nitrogen starvation [20].

The phenotypic similarities between the *acyA^−^* and the *wcoA^−^* mutants do not apply to carotenoid biosynthesis, which was not significantly affected in the latter [20]. As the only predicted WC-1-like photoreceptor in the *Fusarium* proteome [17], the WcoA protein was presumed to mediate photocarotenogenesis in this fungus. However, the *wcoA* ^−^ mutants conserved the photoinduction of the carotenoid pathway, either on carotenoid content or on mRNA levels for the enzymatic genes. Unexpectedly, carotenoid biosynthesis was significantly enhanced in the dark and reduced in the light in the Δ*acyA* mutants. The lower amount of carotenoids in the light is not explained by a lower photoinduction of the *car* genes, but we cannot discard other alternative explanations, e.g., lower mRNA translation of *car* mRNAs, reduced biosynthetic activity or half-life of carotenogenic enzymes, lower capacity for carotenoid storage or higher carotenoid degradation rate. The available information is insufficient to propose an experimentally based hypothesis. On the other hand, the correlation of the enhanced synthesis in the dark with increased mRNA levels suggests a role for cAMP in the transcriptional repression of the *car* genes under these conditions, in which the CarS regulatory protein must play a central role. The identification of the CarS protein, currently in progress, should be a significant contribution in the understanding of this regulatory connection. Interestingly, adenylyl cyclase activity is induced by light in *Trichoderma viride* [41], a fungus in which conidiation is stimulated by light and by nutrient deprivation in darkness. More recently, a model was proposed in this fungus in which cAMP activates indirectly its WC complex (Blr1/Blr2) through the activation of the protein kinase PKA [42]. Such mechanism could explain the similar reduction in conidiation exhibited by the *acyA^−^* and *wcoA^−^ F. fujikuroi* mutants. It must be noted, however, that conidiation is not stimulated by light either in *F. fujikuroi* [20] or in *F. verticillioides* [43].

It was formerly shown that cAMP plays a role in fungal responses against abiotic stress. In the investigated *Fusarium* species, *F. verticillioides* and *F. proliferatum*, loss of adenylyl cyclase resulted in enhanced resistance to heat shock and oxidative stress [23], [24]. In addition, the mutants of G protein subunits of *F. oxysporum* exhibited a reduced cAMP level and enhanced resistance to heat stress [44], [45]. We found that *F. fujikuroi* is highly resistant to heat shock; even though, the Δ*acyA* mutants were able to survive to heat shock treatments that were lethal for the wild type. However, in contrast to *F. proliferatum* and *F. verticillioides*, the Δ*acyA* mutants of *F. fujikuroi* became more sensitive to H_2_O_2_ than the control strain. This result suggests a positive regulatory role of cAMP signaling in oxidative stress resistance in this fungus. The higher sensitivity to H_2_O_2_ of the mutants could also be an indirect effect of its slower growth, but their total incapacity to grow at high H_2_O_2_ concentrations makes this interpretation unlikely. The disparity on oxidative stress resistance with other *Fusarium* species supports the high versatility of the cAMP signaling system, adapted in different manners by closely related fungi. The phenotypic differences are particularly striking if they apply to closely related *Fusarium* species, even more if we consider that heat shock and oxidative stress responses are controlled by at least partially overlapping signaling routes in yeast cells [46]. Experimental results in *F. verticillioides* showed that homologs of yeast HSP26 and GSY2 proteins are up-regulated in the adenylyl cyclase mutant under heat stress. However, the poor overall conservation of transcriptional regulators lying downstream of central components of cAMP-PKA pathway in filamentous species [47] opens the possibilities to other regulatory scenarios.

The different ability of the *F. fujikuroi* and *F. proliferatum* mutants to invade tomato fruit tissue provides another example of differences in the phenotypic effects produced by adenylyl cyclase gene mutation in closely related *Fusarium* species. In contrast to the huge information available on the pathogenicity mechanisms of the tomato pathogen *F. oxysporum*, *f. sp. lycopersici* [48], little is known on the pathogenicity process of *F. fujikuroi*. Actually, it is unclear if the growth on tomato in our test is the result of a pathogenic association or a saprotrophic interaction. *F. oxysporum* is a vascular pathogen [49], and tomato fruit invasion is not its usual colonization mechanism [48]. Nevertheless, it has been successfully used as a fast pathogenicity test in this fungus [48], [54]. The reason is that such invasion needs the secretion of a battery of lytic enzymes, including those degrading plant cell walls. It is plausible that a similar mechanism is involved in the invasion of tomato tissue by its close relatives *F. fujikuroi* and *F. proliferatum*.

Overall, the pleiotropic nature of the cAMP-PKA pathway in closely related *Fusarium* species, i.e. *F. proliferatum F. verticillioides* and *F. fujikuroi*, suggests that stress signaling has been exposed to rapid evolution to tune stress responses in a niche-specific manner, independently of phylogenetic position of the species [47]. Recent data showing significant variations in the resistance to different abiotic stressors by the three *Fusarium* species whose genome sequences are available [50] reinforce this conclusion.

## Materials and Methods

### Strains and culture conditions

The wild type strain, FKMC1995 (*Gibberella fujikuroi* mating population C) was generously provided by J.F. Leslie (Kansas State University Collection, Manhattan, KS, USA). Wild type and *acy1* mutants of *F. proliferatum* were previously described [23]. To obtain conidia, the *F. fujikuroi* strains were grown on CG media (10 g l^−1^ D+(−)glucose, 0.1 g l^−1^ NH_4_NO_3_, 1 g l^−1^ KH_2_PO_4_, 0.5 g l^−1^ MgSO_4_·7H_2_O, and 16 g l^−1^ agar) for 1 week under continuous illumination in a 22°C chamber. cAMP (dibutyryladenosine 3′,5′-cyclic monophosphate sodium salt, Sigma, Saint Louis, MO) was dissolved in milliQ water, sterilized by filtration and added to autoclaved medium (precooled to ca. 50°C) at the desired concentration. Conidia were collected from the surface of the cultures with water, filtered through sterile Whatman paper and counted in a hemocytometer.

For analysis of growth, morphology, carotenoid production, conidiation and response to cAMP addition, the strains were grown at 30°C on DG media [51] with 3 g l^−1^ asparagine instead of NaNO_3_ (DGasn medium). Light incubations were done below a battery of four fluorescents Philips TL-D 18W/840 at a distance of 58 cm, providing *ca.* 5 W m^−2^ white light. For analysis of red pigment production, the strains were cultured in DGasn (also termed high nitrogen agar) or DGasn with 0.3 g l^−1^ asparagine (low nitrogen agar). For gibberellin analysis, experiments were carried out in high nitrogen broth or low nitrogen broth (ICI or 10% ICI liquid media, respectively [52]). Conidiation rates were checked on DGasn media.

For *carB* and *carRA* gene expression analyses, 15 Ø cm Petri dishes with 80 ml of DGasn medium were inoculated with 10^6^ fresh conidia of each strain and incubated in the dark at 30°C during three days. For *gibB* gene expression analyses, 500 ml Erlenmeyer flasks containing 250 ml of high nitrogen broth were inoculated with 10^6^ conidia and incubated for 3 days at 30°C on an orbital shaker at 150 rpm. Then, mycelia were filtered through sterile Whatman paper, washed with sterile distilled water and resuspended in 250 ml of a 2% glucose solution supplemented with microelements (“nitrogen-free solution”, [16]). To compensate for different growth of control strains and Δ*acyA* mutants, three flasks of each mutant were harvested and transferred together to a single flask with the nitrogen-free solution; the final mycelial density was similar to that of the wild type.

### Growth and conidiation

Conidiation analyses were done incubating 50 symmetrically distributed colonies on DGasn Petri dishes at 22°C for 7 days. Conidia determinations were achieved following Prado *et al.* [53].

For growth assays, glass race tubes (17 cm length, 1.6 cm inner width) were filled with 15 ml of DGasn agar. *Ca*. 1 mm mycelial pieces were set at the opposite extremes of the tubes, and cultured under 100% humidity in a 30°C chamber. Growth was measured after 21 days.

Tomato invasive growth assays were performed as described by Di Pietro *et al.* [54]. Tomato fruits were inoculated with 10^6^ fresh conidia of each strain and incubated at 30°C under 100% humidity during 7 days.

### Stress treatments

For heat stress treatment, serial conidia-dilution assays were applied as described elsewhere [55]. Spots of conidial suspensions (5 µl from ten-fold serial dilution series: 2×10^4^–2×10^7^ conidia ml^−1^) of *acyA* ^+^ and Δ*acyA* strains were plated onto DG agar. Heat stress treatments, 1 hour at 42°C, 45°C, 50°C, 55°C or 60°C, were achieved immediately or after four-hour pre-germination at room temperature (RT). Plates were photographed after 2 or 3 days incubation at RT. H_2_O_2_ sensitivity was assessed by three different methods. The serial dilution assay described above was also applied to this stressor at 1–10 mM H_2_O_2_ concentration. H_2_O_2_ sensitivity was also tested by incubating non-germinated conidia (10^6^ cell ml^−1^) in liquid DG media containing 5 mM H_2_O_2_ for 0–2 h at 25°C. Aliquots of the samples were plated onto complex media (CM) agar, incubated for two days at RT and colonies were counted. Percent survival was calculated by dividing the number of colonies obtained after exposures to the H_2_O_2_ treatments by the number of colonies developed from untreated samples. H_2_O_2_ and heavy metal sensitivities were tested by inoculating culture blocks onto DG agar amended with 50–100 mM H_2_O_2_, 3–8 mM CuS0_4_·5H_2_O_2_ or 0.1–1.0 mM CdS0_4_·8/3H_2_O as described earlier [23]. Percent radial growth inhibition was expressed by dividing the colony diameter of the fungus strain grown on abiotic stressor-treated plates by the colony diameter of the same strain grown on non-treated control plates. These experiments were run in triplicate and repeated at least twice.

### Transformation and molecular biology techniques

To obtain the Δ*acyA* mutants, a plasmid was constructed with a hygromycin resistance cassette surrounded by two internal *acyA* segments. For this purpose, a 4.5 kb *acyA* segment containing the catalytic adenylyl cyclase domain was obtained by PCR with primers 1 (5′-ACCCTTACTCATGCAGAAGC-3′) and 2 (5′-ACAACTCTTGCTCTGTGTCG-3′) and cloned in pGEM-T. The resulting plasmid was digested with *Bam*HI, which has two cutting sites in the *Fusarium acyA* segment and no cutting sites in the vector. The digestion removes an internal 1.98 kb segment of the *acyA* gene, which includes most of the catalytic domain, which was replaced with a HygR cassette obtained from plasmid pAN7-1 [56], to give plasmid pDAc. Transformation was done following Proctor *et al.* [57], incubating FKMC1995 protoplasts obtained from 2×10^8^ conidia with 30 µg of plasmid pDAc previously linearized with *Not*I.

The presence of wild type or mutated Δ*acyA* alleles in the transformants was checked by PCR with primers 3 (5′-GATTCTGGGATGTCATCAAGG-3′) and 4 (5′-TATTCCTTTGCCC TCGGACG-3′) were used against primer 2. Primers 1, 2 and 3 were deduced from conserved sequences of the adenylyl cyclase genes in the available *Fusarium* genome sequences, and primer 4 corresponds to an internal segment of the HygR cassette (Fig. 1A).

PCR reactions were carried out with the Expand High Fidelity PCR System (Roche, Sant Cugat del Vallés, Spain). Initial denaturation was at 94°C for 2 min, followed by 35 cycles of 15 s at 94°C, 30 s at 53°C and N s at 68°C (N = 330 s in PCR 3-2 in Fig. 1A, 400 s in PCR 4-2 or 320 s in the PCR to clone the 4.5 kb *acyA* segment), and a final elongation step at 68°C for 10 min.

Southern blot analyses and other molecular biology techniques were achieved following Sambrook et al. [58]. The nylon membranes were probed with the *Bam*HI 1.98 kb segment of the *acyA* gene containing most of the catalytic domain, or the 1.02 kb *hph* coding sequence (Fig. 1A), obtained by PCR with primers 5′-TGCCTGAACTCACCGCGACG-3′ and 5′-TATTCC TTTGCCCTCGGACG-3′. ^32^P-labeled probes were prepared by standard random oligomer priming.

### Gene expression analyses

The mRNA levels of genes *carB*, *carRA* and *gibB* in total RNA samples were determined by real time RT-PCR. For *carB* and *carRA* analyses, 3-day-old samples of submerged mycelia were recovered from the 15 Ø cm Petri dishes before or after 30 or 60 minutes illumination. Mycelia were dried on filter paper, frozen in liquid nitrogen and stored at −80°C. For *gibB* expression, 10 ml mycelial samples were taken from the 3-day-old Erlenmeyer flasks after 0, 6, and 16 h incubation in the nitrogen-free solution in the dark, filtered, frozen in liquid nitrogen and stored at −80°C.

Total RNA samples were obtained with the RNeasy Plant Mini Kit (Qiagen, Chatsworth, CA, USA) and their concentrations were estimated with a Nanodrop ND-1000 spectrophotometer (Nanodrop Technologies, Wilmington, DE, USA). Total cDNA was synthesized with the Transcriptor First Strand cDNA Synthesis kit (Roche, Mannheim, Germany) and stored at −20°C until use. RT-PCR analyses were performed with a LightCycler 480 Real-Time PCR Instrument (Roche) using the LightCycler 480 SYBR Green I Master (Roche). The primers were chosen with the software Primer Express v2.0.0 (Applied Biosystems) from exon sequences of each gene and synthesized (HPLC grade) by StabVida (Oeiras, Portugal). Gene and primers were: *carB*, 5′-TCGGTGTCGAGTACCGTCTCT-3′ and 5′-TGCCTTGCCGGTTGCTT-3′; *carRA*, 5′-CAGAAGCTGTTCCCGAAGACA-3′ and 5′-TGCGATGCCCATTTCTTGA-3′; *gibB*, 5′-TGTCAGCGAATCTGCTCCAA-3′ and 5′-GACGCATAACGGATGAAATGAG-3′. The results for each gene were normalized to the corresponding results obtained with mRNA of the tubulin gene *tubA*, using the primers 5′-CCGGTGCTGGAAACAACTG-3′ and 5′-CGAGGACCTGGTCGACAAGT-3′. The mRNA data obtained with each sample were referred to the value obtained with wild-type mycelia collected before illumination in the case of *carRA* and *carB*, or before incubation in the nitrogen-free solution in the case of *gibB*. Each RT-PCR analysis was performed twice using cDNA samples obtained from two independent experiments.

### Chemical determinations

Carotenoids were extracted from *ca.* 0.05 g freeze-dried samples using a Fastprep-24 device (MP Biomedicals, Irvine, CA). Total amounts of polar and non-polar carotenoids were determined from maximal absorption spectra in hexane according to Arrach *et al.* [59].

For gibberellin determinations, the medium was separated from the mycelia by filtration and 1 ml medium samples were acidified to pH 2.5 with 10 M HCl and passed through Millipore 0.22-µm-pore filters. Gibberellins were extracted as described by Bhalla *et al.* [60] and run in an Hewlett Packard Series 1100 Chromatographer (Agilent Technologies, Palo Alto, CA) equipped with a G1322A degasser, a G1311 quaternary pump, and a G1315A diode array detector. After filtering, samples were vacuum-dried and re-dissolved in 60 µl HPLC grade acetonitrile. 10 µl aliquots were injected through an analytic ProntoSIL Spheribond ODS octadecyl-silyl column (5 µm particle diameter; 250×4.6 mm; Bischoff Chromatography, Leonberg, Germany). Acetonitrile and acidic water (0.01% H_3_PO_4_) in the ratio of 60∶40 was used as mobile phase with a flow rate of 0.6 ml min^−1^. Concentrations were obtained from absorption peaks at 206 nm, using commercial GA3 (Sigma) as a standard.

Thin Layer Chromatography (TLC) analyses of gibberellins were performed extracting 500 µl of acidified filtrate [60]. Extracted samples were vacuum-dried, re-dissolved in 2 µl acetonitrile and run on a Silica gel 60 TLC plastic sheet (Merck, Darmstadt, Germany) using as mobile phase benzene∶n-butyl alcohol∶acetic acid (60∶30∶10). After five minutes, the TLC sheet was air-dried, sprayed with a mixture of ethanol and concentrated sulfuric acid (95∶5) and incubated 5 minutes at 80°C. Developed spots of gibberellic acid were visualized under 302 nm UV.

To quantify production of the red pigment, 15 days old mycelia samples were collected from high and low nitrogen agar cultured at 30°C in the dark to minimize carotenoid biosynthesis, frozen and vacuum-dried. Samples of 0.05 g were extracted in chloroform using a Fastprep-24 device (MP Biomedicals) until bleaching of the reddish pigmentation. The chloroform fractions were vacuum-dried and resuspended in an appropriate volume of the same solvent for spectrophotometric determinations. Amounts of the red pigment were given as absorbance units.

TLC analysis of red pigments were carried out vacuum-drying 200 µl of the sample extracted from the wild type and Δ*acyA* strain T2. The samples were re-dissolved in 2 µl chloroform and run on a Silica gel 60 TLC plastic sheet (Merck, Darmstadt, Germany) using as mobile phase chloroform∶acetone∶formic acid (84∶15∶1). A sample purified from a culture of the bikaverin overproducing strain SG1 [16] was used as a bikaverin standard.

## Supporting Information

Figure S1 **Lack of effect of the Δ** ***acyA*** **mutation on growth in the presence of metals.** Effect of 1 mM Cd or 3 mM Cu on radial growth of the *acyA* ^+^ and Δ*acyA* strains on DGasn agar plates.
(PDF)
