# Supplementary material for: Adenylyl Cyclase Plays a Regulatory Role in Development, Stress Resistance and Secondary Metabolism in Fusarium fujikuroi
Source: PLoS One. 2012 Jan 26;7(1):e28849. doi: 10.1371/journal.pone.0028849 (PMC3266886; doi:10.1371/journal.pone.0028849)

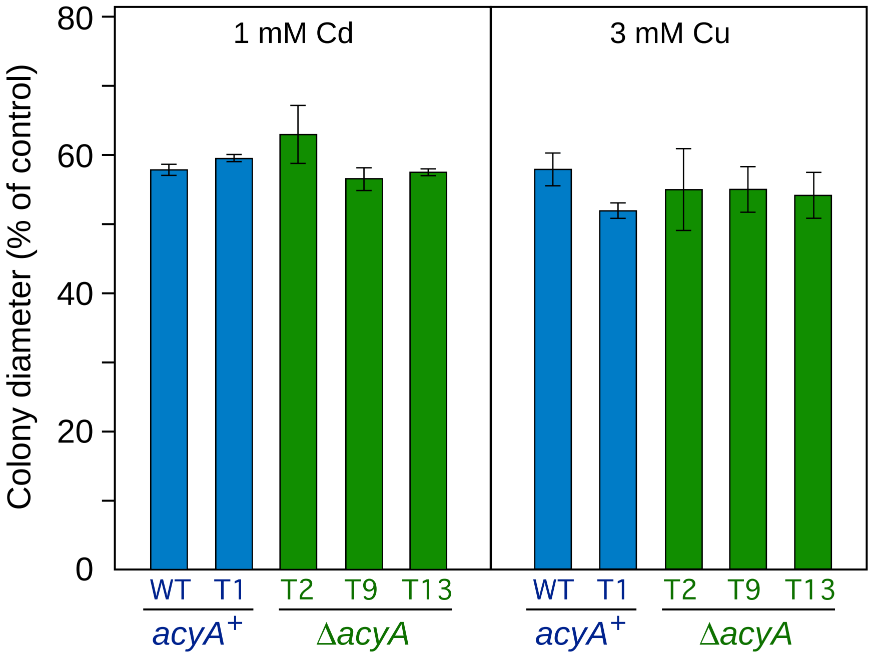

Figure S1. Lack of effect of the Δ*acyA* mutation on growth in the presence of metals. Effect of 1 mM Cd or 3 mM Cu on radial growth of the *acyA*$^{+}$ and Δ*acyA* strains on DGasn agar plates.

Silvestre 4287

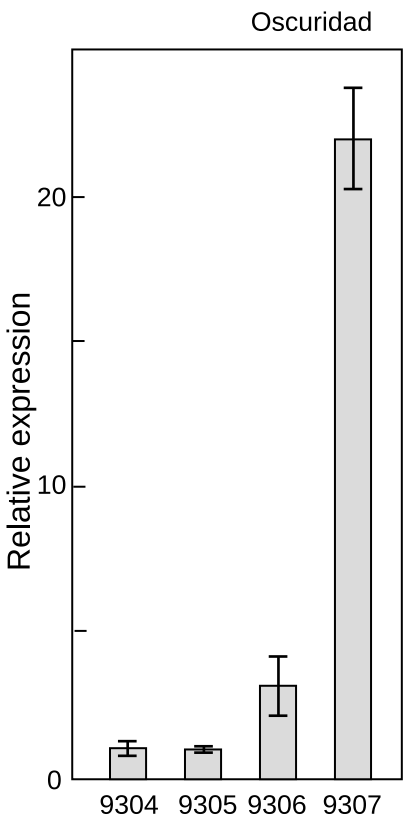

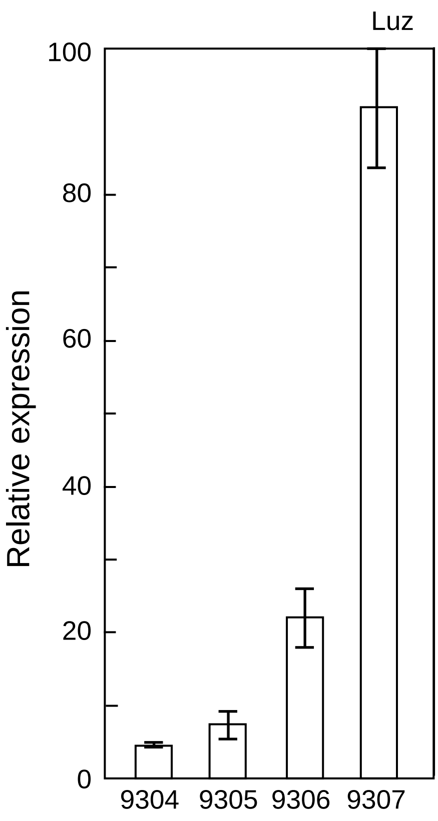

Supplement: Figure S1 — Lack of effect of the Δ acyA mutation on growth in the presence of metals. Effect of 1 mM Cd or 3 mM Cu on radial growth of the acyA + and ΔacyA strains on DGasn agar plates. (PDF) [file pone.0028849.s001.pdf]
